# Heparanase Activates Antithrombin through the Binding to Its Heparin Binding Site

**DOI:** 10.1371/journal.pone.0157834

**Published:** 2016-06-20

**Authors:** Nataliya Bohdan, Salvador Espín, Sonia Águila, Raúl Teruel-Montoya, Vicente Vicente, Javier Corral, Irene Martínez-Martínez

**Affiliations:** 1 Servicio de Hematología y Oncología Médica, Hospital Universitario Morales Meseguer, Centro Regional de Hemodonación, Universidad de Murcia, IMIB-Arrixaca, Murcia, Spain; 2 Grupo de investigación CB15/00055 del Centro de Investigación Biomédica en Red de Enfermedades Raras (CIBERER), Instituto de Salud Carlos III (ISCIII), Madrid, Spain; University of Patras, GREECE

## Abstract

Heparanase is an endoglycosidase that participates in morphogenesis, tissue repair, heparan sulphates turnover and immune response processes. It is over-expressed in tumor cells favoring the metastasis as it penetrates the endothelial layer that lines blood vessels and facilitates the metastasis by degradation of heparan sulphate proteoglycans of the extracellular matrix. Heparanase may also affect the hemostatic system in a non-enzymatic manner, up-regulating the expression of tissue factor, which is the initiator of blood coagulation, and dissociating tissue factor pathway inhibitor on the cell surface membrane of endothelial and tumor cells, thus resulting in a procoagulant state. Trying to check the effect of heparanase on heparin, a highly sulphated glycosaminoglycan, when it activates antithrombin, our results demonstrated that heparanase, but not proheparanase, interacted directly with antithrombin in a non-covalent manner. This interaction resulted in the activation of antithrombin, which is the most important endogenous anticoagulant. This activation mainly accelerated FXa inhibition, supporting an allosteric activation effect. Heparanase bound to the heparin binding site of antithrombin as the activation of Pro41Leu, Arg47Cys, Lys114Ala and Lys125Alaantithrombin mutants was impaired when it was compared to wild type antithrombin. Intrinsic fluorescence analysis showed that heparanase induced an activating conformational change in antithrombin similar to that induced by heparin and with a *K*_*D*_ of 18.81 pM. In conclusion, under physiological pH and low levels of tissue factor, heparanase may exert a non-enzymatic function interacting and activating the inhibitory function of antithrombin.

## Introduction

Heparanase is an endoglycosidase able to cleave heparan sulphate side chains at a limited number of sites, yielding heparan sulphate fragments of still appreciable size (~5–7 kDa) [1–3]. Heparanase is expressed as an enzymatically inactive proheparanase that is later converted into an active enzyme following processing by proteases, such as cathepsin-L [4]. Its activity correlates with the metastatic potential of tumor cells, attributed to enhanced cell dissemination as a consequence of heparan sulphate cleavage and remodeling of the extracellular matrix barrier [5,6]. Heparanase is secreted by platelets after degranulation, leukocytes or endothelial cells and it has multiple roles. It can aid cell invasion by degrading heparan sulphate proteoglycans and can release growth factors bound to heparan-sulphate that initiate angiogenesis, such as VEGF, or activate tissue repair by releasing FGF. Heparan sulphate-disaccharides liberated by heparanase also inhibit TNF production by macrophages with direct consequences as a negative regulator of inflammation [4]. Up-regulated expression of heparanase has been noted in essentially all human tumors examined, as well as in inflammation, wound healing, and diabetic nephropathy [5–7].

Besides, heparanase has been recently revealed as an important modulator of blood coagulation. Heparanase is able to activate tissue factor (TF) in a non-enzymatic manner, and up-regulates TF expression [8]. Heparanase also interacts with tissue factor pathway inhibitor (TFPI) on the cell surface, leading to dissociation of TFPI from the cell membrane of endothelial and tumor cells, resulting in decreased anticoagulant capacity at the cell surface [9]. Antithrombin is the main inhibitor of the coagulation system and its main targets are FXa and FIIa or thrombin [10]. The binding of glycosaminoglycans to the heparin-binding site causes a conformational transition in antithrombin that closes the β-sheet A, which expels the reactive center loop, activating the molecule [11]. This control allows a slight delay in the antithrombin anticoagulant function and restrains it to the site of vascular injury. Therefore, alteration of heparin affinity results in a defective inhibitory function of this serpin. Several studies have reported the procoagulant state derived from mutations affecting the antithrombin heparin binding [12–15].

Under physiological conditions, heparanase is not over-expressed and it does not exert endoglycosidase activity. However, it has been demonstrated that there is an increased heparanase procoagulant activity in healthy shift working nurses in comparison to healthy daytime working nurses [16]. In this study we have addressed the potential effect of heparanase on antithrombin inhibitory function under physiological pH in order to better know its role in coagulation.

## Materials and Methods

### Materials

Low molecular weight heparin (LMWH; Bemiparin) and unfractionated heparin (UFH) were from Rovi (Madrid, Spain). FXa was from Enzyme Research Laboratories (Swansea, United Kingdom) and FIIa was from (Merck Millipore, Madrid, Spain). Culture media were obtained from Gibco (Fisher Scientific, Madrid, Spain).

Recombinant human active heparanase was from R&D systems. TFPI presence on heparanase preparation was evaluated by silver staining and immunostaining with anti-human TFPI (from rabbit, Santa Cruz Biotechnologies, USA) followed by donkey anti-rabbit IgG–horseradish peroxidase conjugate (GE Healthcare, Barcelona, Spain), with detection via an ECL kit (GE Healthcare, Barcelona, Spain). Plasma pooled from healthy volunteers was used as control for TFPI detection. Plasma samples were procured from the donation centre “Centro regional de Hemodonación” (http://www.murciasalud.es/crh.php?iddoc=196989&idsec=202#).

According to R&D, heparanase was purchased after purification by Ni2+-affinity chromatography and removal of the His-tag of recombinant heparanase by protease treatment. This was even proved by checking the presence of histidines on heparanase preparation via immunodetection with mouse anti-histidine (Fisher Scientific, UK) followed by sheep anti-mouse IgG-horseradish peroxidase conjugate (GE Healthcare, Barcelona, Spain), with detection via an ECL kit (GE Healthcare, Barcelona, Spain). Antithrombin with a histidine tag at C-terminal was used as control for histidine detection.

### Proheparanase expression in 293T cells and evaluation of the activation of antithrombin

Proheparanase expression was conducted by transfecting 293T cells with HPSE (Myc-DDK-tagged)-Human heparanase plasmid (Origene, Madrid, Spain).We transfected 340 ng of plasmid for 30 minutes in OptiMEM with LTX (Invitrogen, Barcelona, Spain), as suggested by the manufacturer. After 6 hours, cells were washed with PBS and conditioned medium was added to the cells. Supernatant was recovered 24 hours after transfection and from cells transfected with an empty plasmid, and both were concentrated 10-fold by tangential ultrafiltration using Visvaspin 2, 10 kDa MWCO (GE Healthcare, Barcelona, Spain). After concentration, proheparanase expression was evaluated by using a monoclonal anti-c-Myc antibody (Clontech, Conda laboratories, Madrid, Spain) in a 10% SDS-PAGE under reducing conditions. Monoclonal mouse anti-IgG (Jackson Immunoresearch, PA, USA) was added as secondary antibody. Activation of the inhibitory effect of antithrombin was assayed by electrophoresis. Briefly, wild-type antithrombin (0.179 μM) was incubated for 15 min at 37°C with 10-fold concentrated supernatant of cells transfected with HPSE (Myc-DDK-tagged)-Human heparanase plasmid and with 10-fold concentrated supernatant of cells transfected with the empty plasmid in medium supplemented with HPSE (0.17 nM final concentration); LMWH (6.6 μM) and FXa (0.2 μM). The final reactions were loaded in SDS-PAGE under reducing conditions and complexes were detected by Western blot with a rabbit anti-human antithrombin (Sigma Aldrich, Madrid, Spain) followed by donkey anti-rabbit IgG-horseradish peroxidase conjugate (GE Healthcare, Barcelona, Spain), with detection via an ECL kit (GE Healthcare, Barcelona, Spain).

### Recombinant expression of wild type and mutant antithrombins

Site directed mutagenesis was performed using the Stratagene QuikChange Site-Directed Mutagenesis kit and the appropriate primers to obtain p.P73L, p.R79C, p.K146A and p.K157A mutants on the pCEP4/AT-pS169A plasmid containing the wild-type cDNA sequence of the human antithrombin molecule but that only produce β-glycoform. This plasmid was generously provided by Dr. J Huntington (CIMR, Cambridge). Human Embryonic Kidney cells expressing the Epstein Barr Nuclear Antigen 1 (HEK-EBNA) were grown to 70% confluence at 37°C, 5% CO_2_, in DMEM with GlutaMAX-I medium (Invitrogen, Barcelona, Spain), supplemented with 5% fetal bovine serum (Sigma-Aldrich, Madrid, Spain). We transfected 200 μg/mL of wild type and mutant plasmids for 30 minutes in OptiMEM with LTX (Invitrogen, Barcelona, Spain), as suggested by the manufacturer. After 24 hours, the cells were washed with PBS and changed to a CD-CHO medium (Invitrogen, Barcelona, Spain) supplemented with 4mM L-glutamine and 0.25 mg/mL Geneticin (Invitrogen, Barcelona, Spain). Cells were grown at 37°C for 10 days. The media was harvested and replaced by a fresh medium every 2 days.

### Protein purification and electrophoretic evaluation

Human plasma α-antithrombin was purified from a pool of 100 healthy subjects by heparin affinity chromatography on HiTrap Heparin columns (GE Healthcare, Barcelona, Spain), using an ÄKTA Purifier (GE Healthcare, Barcelona, Spain) in 50 mM Tris-HCl, pH 7.4 in a gradient from 0 to 3 M NaCl. Fractions with antithrombin were applied to a HiTrap Q column (GE Healthcare, Barcelona, Spain). Finally, proteins were eluted using a gradient from 0 to 1 M NaCl and desalted using dialysis tubing (Thermo Fisher, Spain). Recombinant antithrombin from media harvests was purified using the same strategy described for plasma antithrombin. Purity of proteins was evaluated by 8% SDS-PAGE by silver staining, as indicated elsewhere [17]. Proteins were stored at -70°C.

### Evaluation of the inhibitory function of antithrombin in presence of heparanase

Anti-FXa and anti-FIIa activities were assayed by incubating wild-type antithrombin (0.232 nM), with heparanase (0.083 nM) and LMWH (0.096 nM) or UFH (0.03 nM) with FXa (2 μM) or FIIa (2 μM), respectively. Hydrolysis of chromogenic substrates (S-2765 for FXa or S-2238 for FIIa) (Chromogenix, Izasa, Spain) was recorded at 405 nm for 10 min in a plate reader.

Recombinant antithrombin variants (300 ng; 0.179 μM) were incubated with FXa (0.2 μM) for 15 min at 37°C. The reaction was carried out with or without previous incubation with heparanase (0.57 nM) for 5 minutes. Reactions with UFH or LMWH (6.6 μM) were performed with wild type antithrombin and FIIa or FXa, respectively, in order to compare the complexes established when antithrombin was activated by heparanase. The final reactions were loaded in SDS-PAGE under reducing conditions and complexes were detected by silver staining.

### Determination of dissociation equilibrium constant (K_D_) for the antithrombin-heparanase interaction

Equilibrium dissociation constants (*K*_*D*_) for the antithrombin-heparanase interaction were determined essentially as described previously [11,18]. Briefly, the change in intrinsic fluorescence of antithrombin (50 nM) upon titration of the heparanase was monitored at 340 nm on a Cary Eclipse spectrofluorometer (Agilent technologies, Barcelona, Spain), with excitation at 280 nm and using bandwidths of 2.5 nm for both excitation and emission. All titrations were carried out at room temperature under physiological ionic strength (*I* = 0.15) in 20 mm Na_2_HPO4, 100 mm NaCl, 0.1 mm EDTA, 0.1% polyethylene glycol 8000, pH 7.4. Fluorescence emission intensity was taken as the average of 100 measurements recorded at 1-s intervals for each addition of heparin. Data were fitted as previously described [11,18].

### Structural modelling

Structural modeling was rendered by using SWISS-MODEL and Swiss-PdbViewer programs[19]. SWISS-MODEL used 3vny.1.A as template of heparanase, since it hadthe higgest homology in sequence, and corresponds to beta-glucuronidase from *Acidobacterium capsulatum*.

## Results

### Antithrombin activation by heparanase

**Fig 1:**
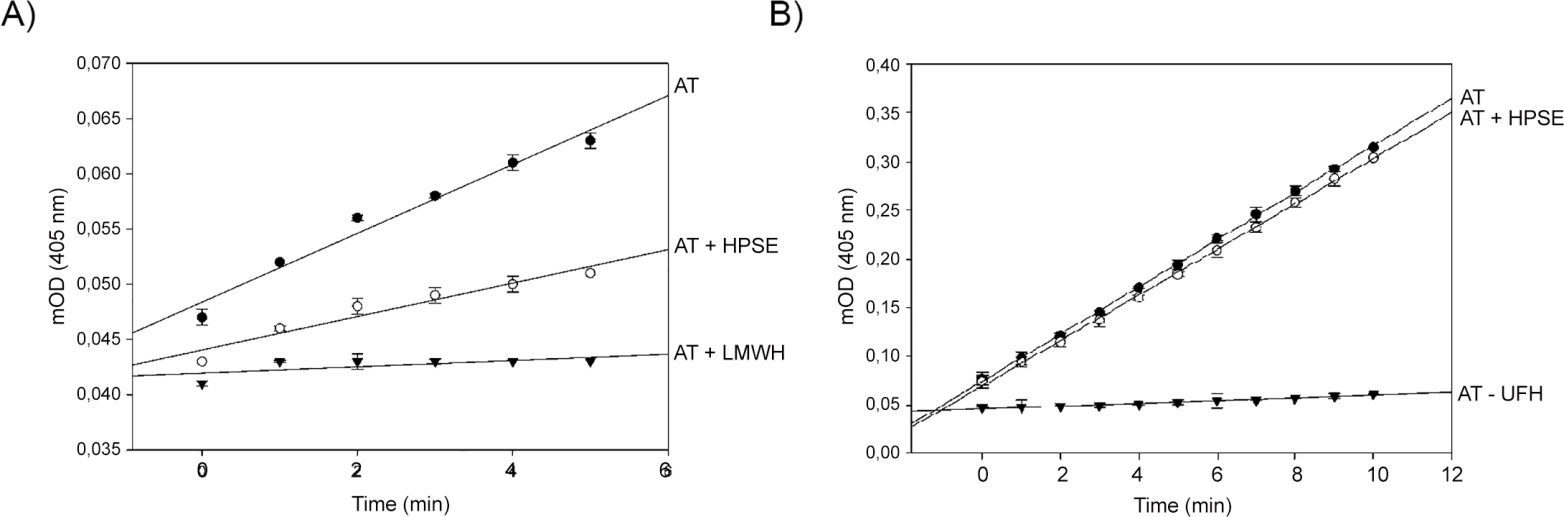
Antithrombin inhibitory activity upon heparanase-mediated activation. Anti-FXa (A) and anti-FIIa (B) activities of antithrombin upon activation with low molecular weight heparin (LMWH) for anti-FXa or unfractionated heparin (UFH) for anti-FIIa, and heparanase (HPSE) evaluated using a chromogenic method in which the hydrolysis of the susbtrates S-2765 (for FXa) or S-2238 (for FIIa) are followed at 405 nm. Measurements were performed in duplicate.

Incubation of antithrombin with heparanase in the absence of heparin provoked the activation of antithrombin in its inhibitory function of FXa (Fig 1A), and slightly increased the inhibitory effect on FIIa (Fig 1B), indicating that there is an allosteric activation of antithrombin. Moreover, heparanase did not impair the activation of antithrombin by LMWH or UFH (data not shown).

Despite heparanase was purified by Ni2+-affinity chromatography instead of a heparin affinity column, the presence of contaminant heparin in the heparanase preparation was the first premise to be addressed. To rule out the presence of heparin, heparanase was incubated overnight at 37°C in 80 mM CH_3_COONa, 250 mM NaCl, pH 4.5, a procedure that activated the endoglycosidase function of heparanase. After this incubation, the preparation was dialysed in 20 mM NaH_2_PO_4_ buffer pH 7.4. The inhibition of FXa by antithrombin upon heparanase-mediated activation was not affected by activation of the endoglycosidase activity of heparanase, giving a result comparable to that shown in Fig 1A. The absence of a histidine tag in the protein was also proved, since protease cleavage was used to remove the tag after purification of the protein by Ni2+-affinity chromatography (S1A Fig).

Potential contamination with TFPI in the heparanase preparation that might explain the inhibition of FXa was also ruled out by the absence of immunologic detection of TFPI (S1B Fig).

### Evaluation of the interaction between antithrombin and heparanase and consequences in the formation of covalent complexes between antithrombin and its target proteases

**Fig 2:**
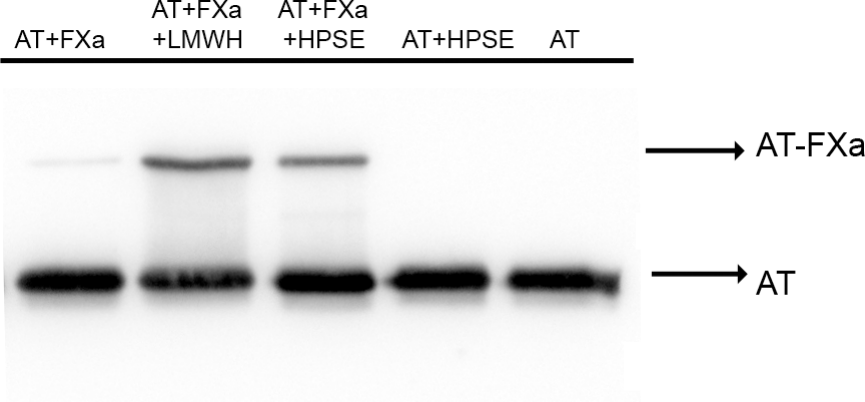
Detection of covalent complexes between antithrombin and FXa upon heparanase-mediated activation. SDS-PAGE under reducing conditions of antithrombin in complex with FXa upon activation with low molecular weight heparin (LMWH) and heparanase (HPSE). Detection was done by western blot.

We firstly evaluated whether the interaction between human plasma antithrombin and heparanase affected the electrophoretic mobility of antithrombin in SDS-PAGE under reducing conditions. As shown in Fig 2, antithrombin and heparanase did not establish covalent interactions. However, accordingly to the functional assays previously described, heparanase exacerbated the formation of covalent complexes between antithrombin and FXa, although with reduced efficiency in comparison to LMWH(Fig 2)

We also addressed whether proheparanase was able to bind and activate antithrombin. Transfected 293T cells with a plasmid containing the sequence of proheparanase secreted this protein to the supernatant (S2A Fig left panel). The effect of proheparanase on the activation of antithrombin and the consequent inhibitory effect of FXa was evaluated by the ability of antithrombin to establish covalent complexes with FXa. Proheparanase activated antithrombin (S2B Fig, right panel).

### Determination of heparanase binding site in antithrombin

**Fig 3:**
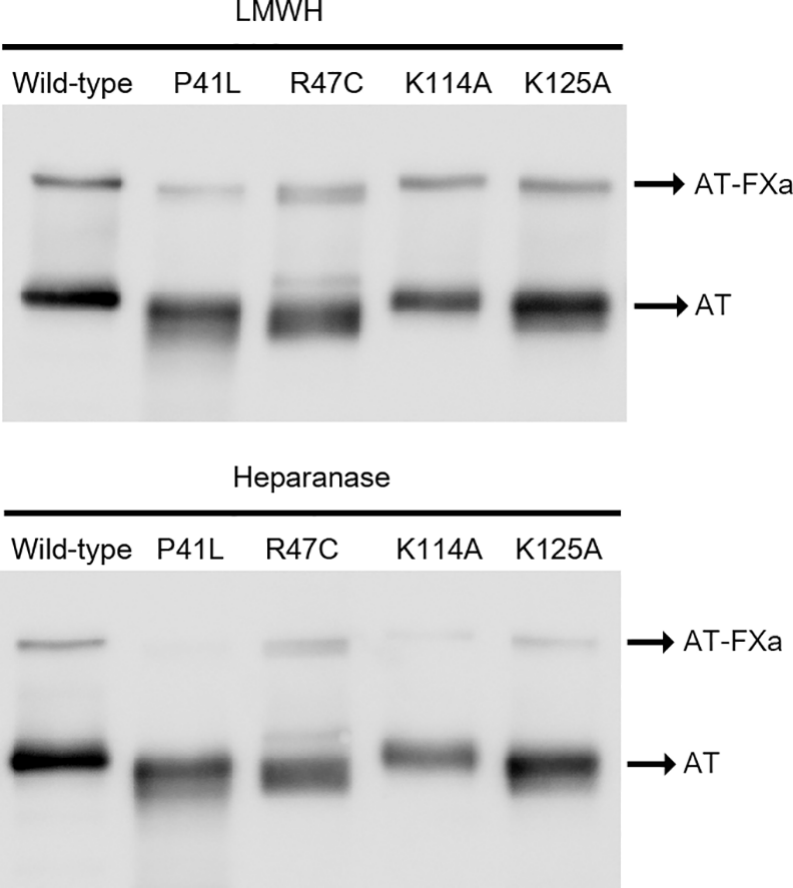
Formation of complex of different antithrombin mutants with FXa upon heparanase-mediated activation. SDS-PAGE under reducing conditions of Pro41Leu, Arg47Cys, Lys114Ala and Lys125Ala variants of antithrombin in complex with FXa upon activation with low molecular weight heparin (LMWH) and heparanase (Hpase). Wild type antithrombin was used as positive control.

As the heparin binding site of antithrombin is well defined[20], we expressed and purified different antithrombin variants with mutations affecting key residues of this domain to determine if they could also be involved in the heparanase binding: Pro41Leu, Arg47Cys, Lys114Ala and Lys125Ala.Electrophoretic evaluation of covalent complexes with FXa in presence of LMWH and heparanase showed that P41L mutant was the most affected protein in the interaction between antihrombin and FXa when antithrombin is activated by heparanase (Fig 3).

Then, to define the affinity of heparanase to antithrombin and to determine the contribution of each one of these residues to such interaction, *K*_*D*_ was calculated through intrinsic fluorescence studies. We firstly checked that heparanase did not emitted fluorescence under the conditions assayed (S3 Fig). As shown in Table 1, Pro41 and Lys125 must be key residues for heparanase binding because the *K*_*D*_ increased when these residues are mutated. Intriguingly, the *K*_*D*_ value of the other two mutants, Arg47Cys and Lys114Ala, also increased in comparison with wild type antithrombin, but the maximum fluorescence increase ((ΔFmax/Fo)*100) was smaller, which means that these residues are affecting the intrinsic fluorescence emission when antithrombin is activated by heparanase.

**Table 1:** Equilibrium dissociation constants for heparanase binding to different variants of antithrombin.

Variant	*K*_*D*_ (pM) ± SD	ΔFmax (Arbitrary units)	Fo	(ΔFmax/Fo)*100
**Wild-Type**	18.81 ± 0.50	18.51	17.30	106.99
**Pro41Leu**	85.41 ± 0.51	18.85	21.91	86.03
**Arg47Cys**	74.09 ± 1.11	6.48	15.44	41.97
**Lys114Ala**	61.78 ± 0.73	6.57	10.78	60.95
**Lys125Ala**	84.69 ± 0.96	21.42	23.48	91.23

Determination of residues involved in the heparanase binding to antithrombin by evaluation of the intrinsic fluorescence emission of antithrombin though heparanase titration. *K*_*D*_ values determine the affinity of antithrombin mutants in comparison to wild type.

## Discussion

Heparanase is a protein with functions that depend on its enzymatic activity. These functions are closely associated with tumor malignancy. Related to its endoglycosidase activity, heparanase may function as a potent modulator of tumor behavior due to its pro-tumorigenic, pro-angiogenic and pro-metastatic activities. Moreover, several studies have demonstratedthat heparanase is up-regulated in all human sarcomas and carcinomas and it is detected at elevated levels in body fluids of breast cancer patients[21]. In addition, heparanase seems to regulate secretion, composition, and function of tumor cell-derived exosomes [22]. On the other hand, heparanase also exerts functions independently of its enzymatic activity. Hence, it has been described that heparanase affects the hemostatic system in a non-enzymatic manner, by directly enhancing TF activity and up-regulating its expression[23]. This results in an increased factor Xa production and leads to dissociation of TFPI from the endothelial membrane surface, thus activating the coagulation system[24]. A potential role of heparanase in coagulation has also been suggested based on the high expression of this protein during the pro-thrombotic state of most neoplasms [23].

**Fig 4:**
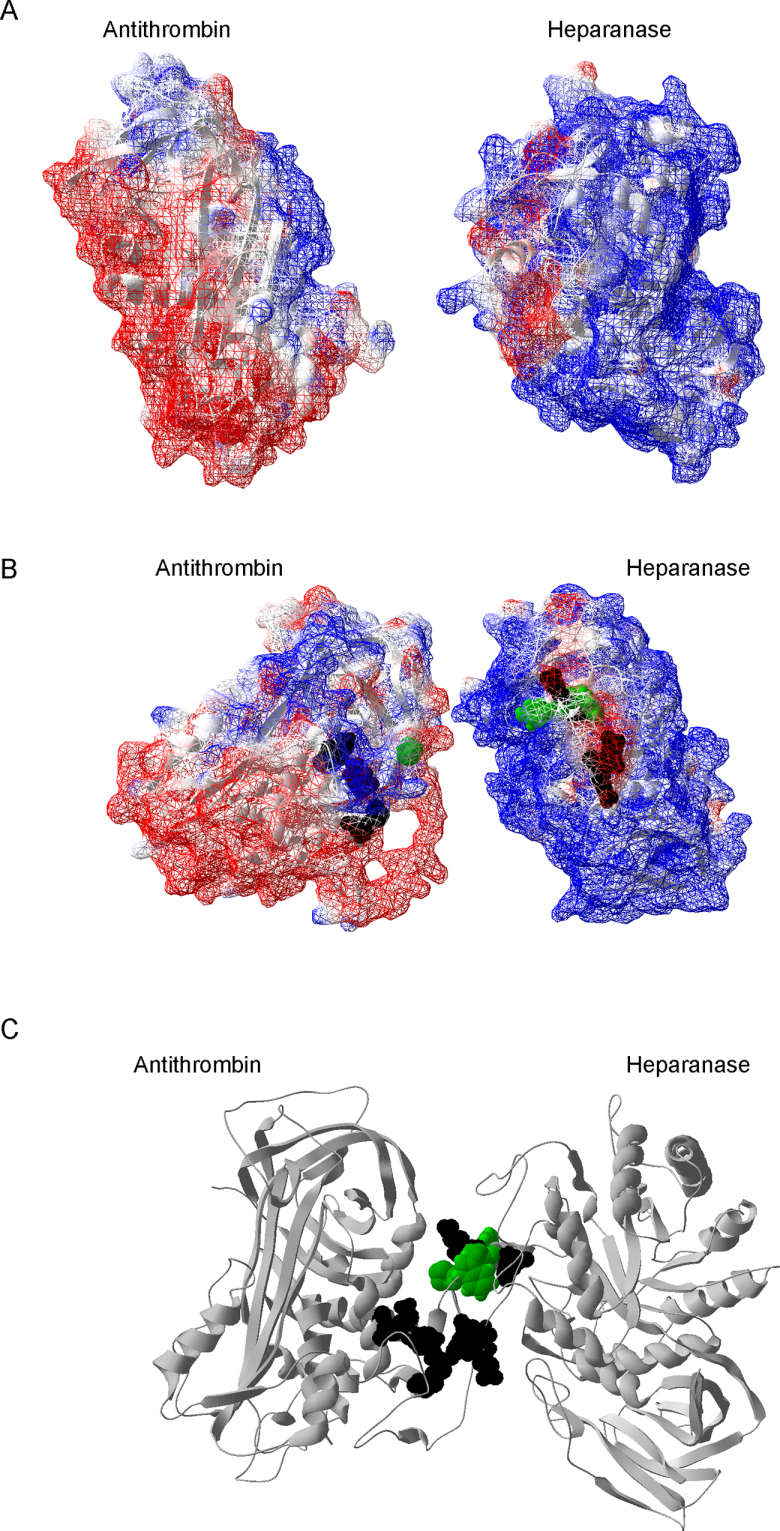
Structural representation of antithrombin and heparanase. A) Molecular surface representation of antithrombin (1t1fA) and the structural model of human heparanase (3vny.1.A). Color blue represents positively charged surface and red displays anionic surface. White is assigned for neutral charged surface. B) Representation of those residues in antithrombin involved in the binding of heparanase and those potentially concerned in heparanase. Residues are displayed with spheres. Those residues potentially involved in electrostatic binding are colored in black spheres (Arg47, Lys114 and Lys125 in antithrombin, and Asp135, Glu137, Glu138, Glu143 and Glu148 in heparanase). Pro41 in antithrombin is highlighted in green. The suggested residues in heparanase (Trp 144 and Tyr146) as involved in the interaction with Pro41 are also colored in green. C) Suggested docking between antithrombin and heparanase.

In our study, we show a new function of heparanase on the hemostatic system that does not require its enzymatic action, and that, therefore, it can be played under physiological conditions, as the physiological pH is not the optimal for the endoglycosidase function [25]. We have demonstrated that antithrombin and heparanase may interact *in vitro* at physiological pH. This interaction did not involve the formation of a covalent complex that is the way in which antithrombin inhibits its target serine proteases[26]. The incubation between both proteins resulted in the activation of antithrombin. The heparanase-mediated activation of antithrombin resulted in an increased inhibition of FXa. This could imply that heparanase-mediated inhibition of FXa by antithrombin happens by an allosteric mechanism[26]. We also analyzed whether proheparanase was able to exert this activating function, as it share many structural domains with the active heparanase [3]. Our results showed that proheparanase was also able to increase the inhibitory effect of antithrombin. Since the activation of antithrombin by heparanase seemed to provoke the same effect as heparins, we presumed that heparanase could bind to the heparin binding domain of antithrombin. Using different mutants with low heparin affinity [15,27], we have also defined some residues in antithrombin that might be relevant for the interaction with heparanase. Except for Pro41, the rest of the residues that we have defined involved in the interaction of antithrombin with heparanase are positively charged. This means that if electrostatic interactions are mediating the binding between both proteins, heparanase should have a negatively charged domain. Based on a structural model, it is shown that, although most of the protein is positively charged, heparanase presents a negatively pocket, also present in proheparanase, consisting of the following residues: Asp135, Glu137, Glu138, Glu143, and Glu148 (Fig 4).

Moreover, Trp144 and Tyr146, which are placed in this pocket, could interact with Pro41 in antithrombin, as it has been described that proline residues preferentially interacts with aromatic residues [28] (Fig 4). Even though heparanase may interact with heparan sulphates, which is indeed favored by the high concentration of positive charges in heparanase and the negative groups in the heparan sulphates, it has been also reported that heparanase is able to bind histidine rich glycoprotein [29]. Thus, it seems that heparanase might interact with other proteins through the negatively charged pocket.

In summary, our study shows that heparanase might have an anticoagulant function under physiological pH; where the endoglycosydase activity is not produced due to the neutral pH. However, the relevance of this function may be scarce, as heparanase has a low expression under these conditions. In those conditions where TF may be highly exposed and levels of heparanase are increased (16), the anticoagulant function mediated by the activation of antithrombin could compensate somehow the activation of the TF expressed. Nevertheless, our study has defined the potential interaction between these two proteins that should be considered when the functions of heparanase are evaluated.

## Supporting Information

S1 Fig Absence of the Histidine-tag and TFPI in the heparanase preparation. SDS-PAGE under reducing conditions and western blot of heparanase sample for immunodetection of the Histidine-tag (left panel) or TFPI (right panel). Antithrombin with a histidine-tag at C-terminal (AT-His) was used as control for the histidine immunodetection (20 ng), to compare with heparanase (20 ng). Control plasma (0.5 μl) from a pool of 100 healthy volunteers was used for TFPI immunodetection. The mean plasma concentration of TFPI in a healthy adult population is 89 ng/ml. Heparanase was loaded at a final concentration of 24 ng. Absence of signal confirms the purity of heparanase preparation, and the absence of histidines indicates that the protein was purified by using N2+-affinity chromatography followed by tag removal with the suitable protease.
(TIF)

S2 Fig Proheparanase expression in the supernatant of 293T cells and effect on the activation of antithrombin. A) Proheparanase expression on the conditioned medium of 293 cells transfected with a plasmid expressing proheparanase Myc-DDK-tagged and an empty plasmid (Mock) was assayed by Western Blot using an anti-c-Myc antibody after SDS-PAGE under reducing conditions. B) Detection of covalent complexes between antithrombin and FXa upon proheparanase and heparanase-mediated activation. SDS-PAGE under reducing conditions of antithrombin in complex with FXa upon activation with low molecular weight heparin (LMWH), active heparanase (HPSE), 10-fold concentrated medium of cells transfected with proheparanase (mProHPSE); 10-fold concentrated medium of cells transfected with an empty plasmid but supplemented with active heparanase (mHPSE). Detection was done by western blot using an anti-antithrombin antibody.
(TIF)

S3 Fig Intrinsic fluorescence change in antithrombin caused by heparanase titration. Black dots represent the conformational activation of antithrombin (+AT). White dots represent the fluorescence of the heparanase added during the titration (-AT).
(TIF)

## Abbreviations

AT: antithrombin
LMWH: low molecular weight heparin
UFH: unfrationated heparin
TF: tissue factor
TFPI: tissue factor pathway inhibitor
