# Supplementary figures and images for: Heparanase Activates Antithrombin through the Binding to Its Heparin Binding Site
Source: PLoS One. 2016 Jun 20;11(6):e0157834. doi: 10.1371/journal.pone.0157834 (PMC4913942; doi:10.1371/journal.pone.0157834)

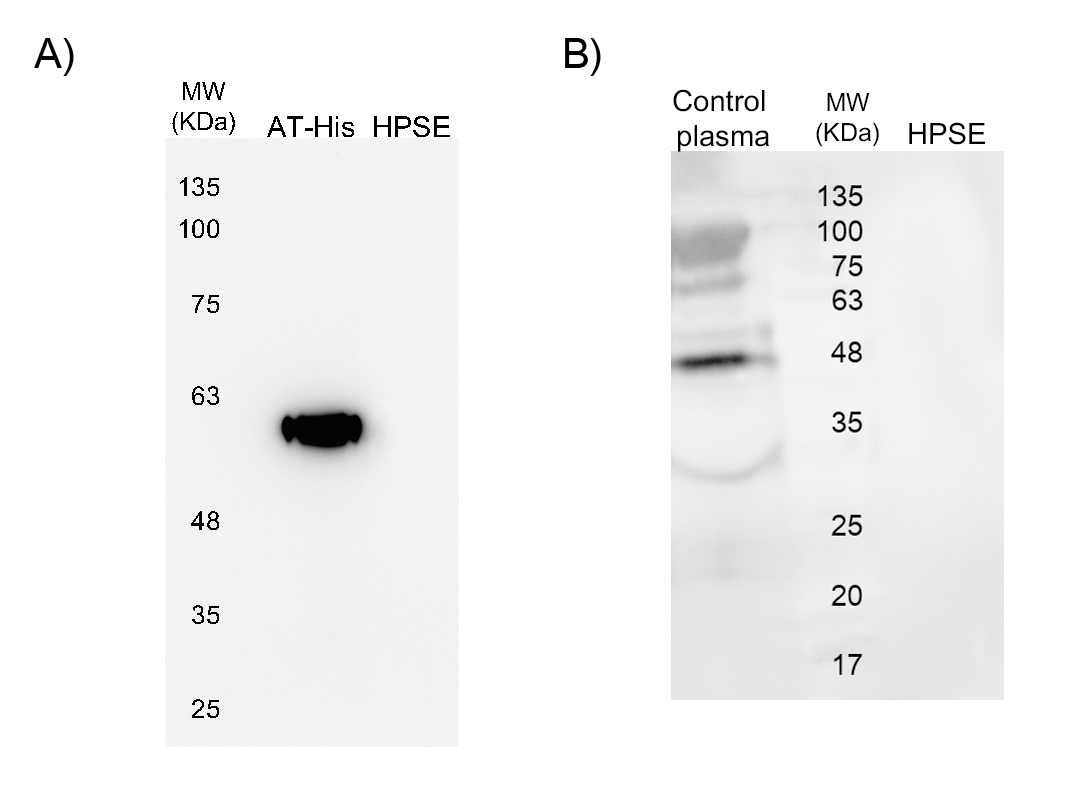

Supplement: S1 Fig — SDS-PAGE under reducing conditions and western blot of heparanase sample for immunodetection of the Histidine-tag (left panel) or TFPI (right panel). Antithrombin with a histidine-tag at C-terminal (AT-His) was used as control for the histidine immunodetection (20 ng), to compare with heparanase (20 ng). Control plasma (0.5 μl) from a pool of 100 healthy volunteers was used for TFPI immunodetection. The mean plasma concentration of TFPI in a healthy adult population is 89 ng/ml. Heparanase was loaded at a final concentration of 24 ng. Absence of signal confirms the purity of heparanase preparation, and the absence of histidines indicates that the protein was purified by using N2+-affinity chromatography followed by tag removal with the suitable protease. (TIF) [file pone.0157834.s001.tif]

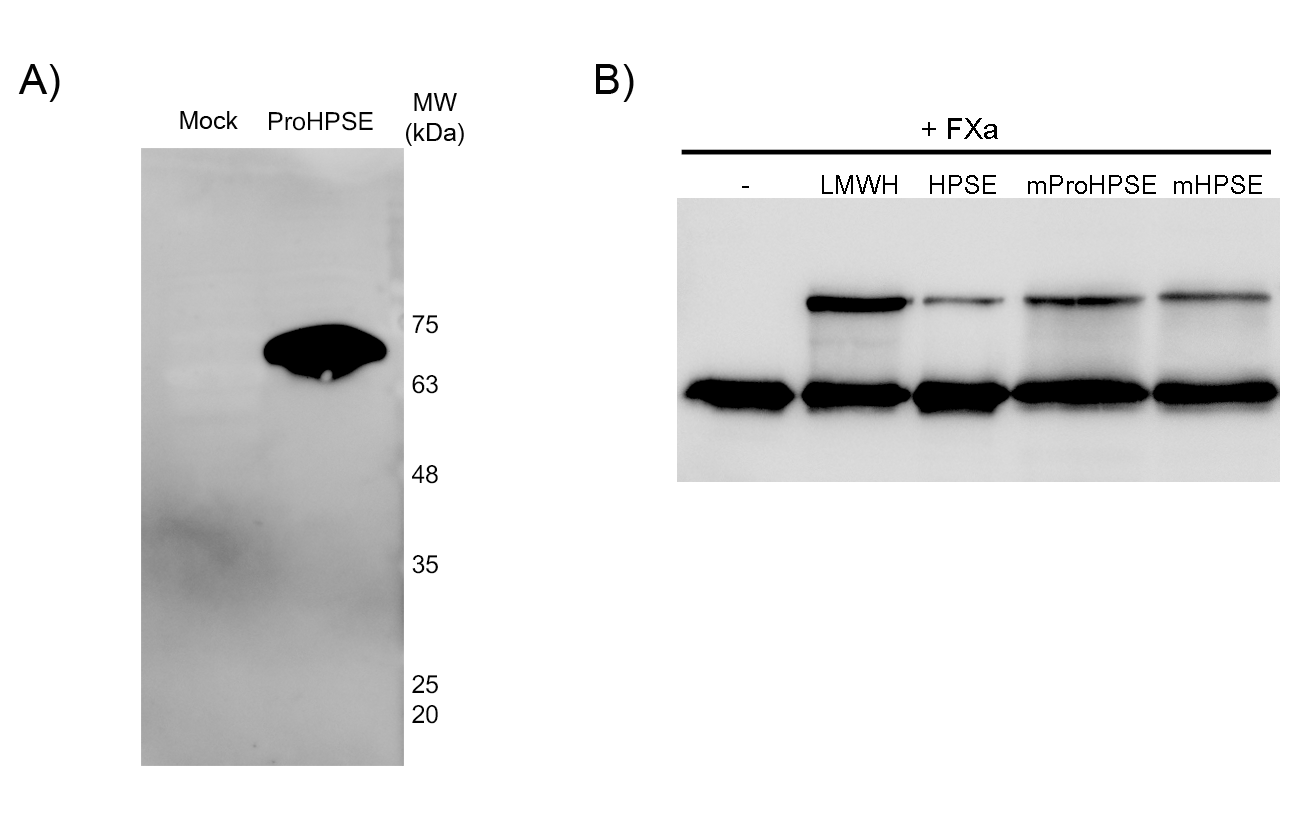

Supplement: S2 Fig — A) Proheparanase expression on the conditioned medium of 293 cells transfected with a plasmid expressing proheparanase Myc-DDK-tagged and an empty plasmid (Mock) was assayed by Western Blot using an anti-c-Myc antibody after SDS-PAGE under reducing conditions. B) Detection of covalent complexes between antithrombin and FXa upon proheparanase and heparanase-mediated activation. SDS-PAGE under reducing conditions of antithrombin in complex with FXa upon activation with low molecular weight heparin (LMWH), active heparanase (HPSE), 10-fold concentrated medium of cells transfected with proheparanase (mProHPSE); 10-fold concentrated medium of cells transfected with an empty plasmid but supplemented with active heparanase (mHPSE). Detection was done by western blot using an anti-antithrombin antibody. (TIF) [file pone.0157834.s002.tif]
